# The pitfalls of using birthweight centile charts to audit care

**DOI:** 10.1371/journal.pone.0235113

**Published:** 2020-06-23

**Authors:** Roshan John Selvaratnam, Mary-Ann Davey, Euan Morrison Wallace

**Affiliations:** 1 Department of Obstetrics and Gynaecology, The Ritchie Centre, Monash University, Melbourne, Victoria, Australia; 2 Department of Health and Human Services, Safer Care Victoria, Victorian Government, Melbourne, Victoria, Australia; University of Cambridge, UNITED KINGDOM

## Abstract

**Objectives:**

Timely delivery of fetal growth restriction (FGR) is important in reducing stillbirth. However, targeted earlier delivery of FGR preferentially removes smaller babies from later gestations, thereby right-shifting the distribution of birthweights at term. This artificially increases the birthweight cutoffs defining the lower centiles and redefines normally grown babies as small by population-based birthweight centiles. Our objective was to compare updated Australian national population-based birthweight centile charts over time with the prescriptive INTERGROWTH-21^st^ standard.

**Methods:**

A retrospective descriptive study of all singleton births ≥34 weeks’ gestation in Victoria, Australia in five two-year epochs: 1983–84, 1993–94, 2003–04, 2013–14, and 2016–17. The birthweight cutoffs defining the 3^rd^ and 10^th^ centile from three Australian national population-based birthweight centile charts, for births in 1991–1994, in 1998–2007, and 2004–2013 respectively, were applied to each epoch to calculate the proportion of babies with birthweight <3^rd^ and <10^th^ centile. The same analysis was done using the INTERGROWTH-21^st^ birthweight standard. To assess change over gestation, proportions were also calculated at preterm, early term and late term gestations.

**Results:**

From 1983–84 to 2016–17, the proportion of babies with birthweight <3^rd^ fell across all birthweight centile charts, from 3.1% to 1.7% using the oldest Australian chart, from 3.9% to 1.9% using the second oldest Australian chart, from 4.3% to 2.2% using the most recent Australian chart, and from 2.0% to 0.9% using the INTERGROWTH-21^st^ standard. A similar effect was evident for the <10^th^ centile. The effect was most obvious at term gestations. Updating the Australian population birthweight chart progressively right-shifted the birthweight distribution, changing the definition of small over time. The birthweight distribution of INTERGROWTH-21^st^ was left-shifted compared to the Australian charts.

**Conclusions:**

Locally-derived population-based birthweight centiles are better for clinical audit of care but should not be updated. Prescriptive birthweight standards are less useful in defining ‘small’ due to their significant left-shift.

## Introduction

Fetal growth restriction (FGR) is a major risk factor for stillbirth [1]. Improving the detection of FGR, allowing for more timely delivery, has been shown to be an effective approach to reducing stillbirth at a population level [2].

However, increasing intervention for FGR is changing the very definition of small, as defined by population-derived birthweight centiles [3], the “yardstick” by which clinical care is audited and hospital performance is assessed [4–7]. This has been shown most recently in Victoria, Australia, where improved detection of FGR has reduced the proportion of babies with birthweight <3^rd^ centile from 3.1% to 1.9% [2]. Over time, this will cause the very birthweight cutoff that defines the 3^rd^ centile to increase. In that same Australian population it has been projected that the birthweight defining the 3^rd^ centile at 40 weeks’ gestation will increase by 150 grams over 35 years [3]. This effect occurs because of the detection and earlier delivery of smaller babies, removing them from the population at term and thereby right-shifting the birthweight distribution. Continued updating of population-derived birthweight centiles will progressively redefine an increasing number of normally grown babies as ‘small’.

One solution is to use prescriptive birthweight charts to audit care. Such charts, including INTERGROWTH-21^st^ [8, 9], WHO [10], and others [11], are derived from healthy populations specifically selected to demonstrate ideal fetal growth applicable across all countries. Theoretically, prescriptive charts offer the benefit of stability over time, not subject to the artefactual drift of updated population-based charts.

With a view to replacing population-based birthweight centile charts with prescriptive charts, we sought to compare the effect of using Australian national population birthweight centile charts over time with the INTERGROWTH-21^st^ birthweight standard to define babies with birthweight <3^rd^ and <10^th^ centile.

## Methods

We undertook a retrospective descriptive study using data from the Victorian Perinatal Data Collection (VPDC), a legislated data collection of every birth in Victoria ≥20 weeks’ gestation. We analysed all singleton births at ≥34 weeks’ gestation in five two-year epochs: 1983–84, 1993–94, 2003–04, 2013–14, and 2016–17. We used two-year epochs to afford sufficient numbers of births at all gestations.

There have been three published Australian national birthweight centile charts for singleton births: for births in 1991–1994 [12], in 1998–2007 [13], and in 2004–2013 [14]. We applied the birthweight cutoff for the 3^rd^ and 10^th^ centile for each gestation and sex in each of the three charts to all five two-year epochs to calculate the proportion of babies born between 34 and 42 weeks’ gestation that would have a birthweight <3^rd^ and <10^th^ centile over time. We performed the same analysis using the INTERGROWTH-21^st^ newborn birthweight standard [15]. Since INTERGROWTH-21^st^ only produced birthweight centiles by completed day, rather than completed week of gestation, we used the birthweight cutoff for the 3^rd^ and 10^th^ centile at the midpoint of each week of gestation as the birthweight cutoff for that completed week. For instance, the birthweight cutoff for the 3^rd^ centile at 40 weeks’ and 3 days was used as the birthweight cutoff for the 3^rd^ centile for all babies born at 40 weeks’.

To assess the change in the proportion of babies with birthweight <3^rd^ and <10^th^ centile at different gestations over time, we grouped babies into those born at preterm (34–36 weeks’), early term (37–39 weeks’), and late term (40–41 weeks’) gestations. We compared the proportion of babies with birthweight below the 3^rd^ and 10^th^ centile in each of these gestation groups over time.

Statistical significance was assessed using chi-square test of proportions. Proportions were compared between the 1983–84 epoch and 2016–17 epoch. A p-value < 0.05 was considered statistically significant (two-tail test).

All analyses were performed using the SPSS statistical package version 24 (IBM Corp., Armonk, New York, USA).

Records with missing birthweights and sex were excluded (both <0.1%).

This study was approved by the Monash University Human Research Ethics Committee (#20461). The data underlying the results presented in the study are available from a third party. Deidentified patient data may be requested for research purposes from: https://vahi.freshdesk.com/support/home.

## Results

The number of singleton births ≥34 weeks’ gestation in Victoria increased from 115,974 in 1983–84 to 152,509 in 2016–17. Table 1 presents population characteristics over time for our study population. Over time, women were more likely to be older, nulliparous, born outside Australia, and identify as Indigenous. Mean gestation and birthweight decreased, while iatrogenic onset of labour increased.

**Table 1:** Population characteristics in each two-year epoch for babies born between 34 and 42 weeks’ gestation.

	1983–84	1993–94	2003–04	2013–14	2016–17
Total (n)	115974	122387	120054	148979	152509
Maternal age (mean (SD))	27.2 (4.8)	28.9 (5.1)	30.4 (5.3)	30.8 (5.5)	31.1 (5.2)
Nulliparous (n (%))	45031 (38.8)	48761 (39.8)	50952 (42.4)	66951 (44.9)	66336 (43.5)
Male (n (%))	59482 (51.3)	63011 (51.5)	61451 (51.2)	76334 (51.2)	78370 (51.4)
Previous stillbirth (n (%))	1974 (1.7)	1580 (1.3)	1281 (1.1)	1772 (1.2)	1847 (1.2)
Gestation (mean (SD)) (weeks’)	39.5 (1.4)	39.4 (1.4)	39.2 (1.4)	39.0 (1.4)	38.8 (1.4)
Birthweight (mean (SD)) (grams)	3420 (493)	3428 (499)	3435 (509)	3413 (504)	3391 (494)
Late preterm (34–36 weeks’) (n (%))	4310 (3.7)	4786 (3.9)	5218 (4.3)	7154 (4.8)	7567 (5.0)
Maternal country of birth (n (%))					
Australia	86940 (75.2)	91731 (75.1)	91259 (76.3)	96372 (65.2)	93279 (61.6)
Not Australia	28629 (24.8)	30407 (24.9)	28795 (24.1)	52607 (35.6)	59230 (39.1)
Indigenous mother (n (%))	547 (0.5)	876 (0.7)	751 (0.6)	1915 (1.3)	2119 (1.4)
Labour onset (n (%))					
Induction of labour	27473 (23.7)	26371 (21.5)	32684 (27.2)	41558 (27.9)	51220 (33.6)
Pre-labour caesarean section	8776 (7.6)	12131 (9.9)	18993 (15.8)	27993 (18.8)	30732 (20.2)

^a^ Missing data excluded so numbers may not add to total

Table 2 describes the major differences between birthweight centile charts.

**Table 2:** Differences between Australian population-based birthweight centile charts and INTERGROWTH-21^st^ birthweight centile chart.

	Roberts et al.	Dobbins et al.	AIHW	INTERGROWTH-21^st^
Years analysed	1991–1994	1998–2007	2004–2013	2009–2013
Published year	1999	2012	2019	2014
Population	National Australian data	National Australian data	National Australian data	NCSS prescriptive subpopulation^a^
Inclusion criteria	Livebirths to Australian-born non-indigenous women	All livebirths	All livebirths	Low risk of fetal growth impairment
Sample size	740847	2528641	2801405	20486
Gestations	20–44	20–44	20–44	33–42
Methodology	Tabulation	Tabulation	Tabulation	Fractional polynomials, LMS, LMST, LMSP methods^b^
Gender specific	Yes	Yes	Yes	Yes

^a^ NCSS (Newborn Cross-Sectional Study), LMS (lambda, mu, and sigma method)

^b^ LMST (lambda, mu, sigma, assuming Box-Cox t distribution), LMSP (lambda, mu, sigma, assuming Box-Cox power exponential distribution)

Table 3 displays the proportion of babies born between 34 and 42 weeks’ gestation in the study epochs classified as birthweight <3^rd^ and <10^th^ centile, by birthweight centile chart. There were substantial differences between the charts, and for each chart, over time.

**Table 3:** Proportion (%) of babies born with birthweight <3^rd^ and <10^th^ centile between 34 and 42 weeks’ gestation in each two-year epoch by birthweight centile chart.

	1983–84	1993–94	2003–04	2013–14	2016–17
	n = 115974	n = 122387	n = 120054	n = 148979	n = 152509
	Proportion with birthweight <3^rd^ centile (%)				
**Roberts et al. (1991–1994)**	3.5	3.1	2.7	2.1	1.7^a^
**Dobbins et al. (1998–2007)**	3.9	3.4	2.9	2.3	1.9^a^
**AIHW (2004–2013)**	4.3	3.8	3.3	2.7	2.2^a^
**INTERGROWTH-21**^**st**^	2.0	1.7	1.5	1.2	0.9^a^
	**Proportion with birthweight <10**^**th**^ **centile (%)**				
**Roberts et al. (1991–1994)**	11.3	10.0	8.9	8.2	7.8^a^
**Dobbins et al. (1998–2007)**	12.4	10.9	9.7	8.9	8.4^a^
**AIHW (2004–2013)**	13.1	11.6	10.3	9.5	9.0^a^
**INTERGROWTH-21**^**st**^	6.8	6.0	5.3	4.8	4.5^a^

^a^ P-values < 0.001 (1983–84 proportion vs. 2016–17 proportion)

Tables 4 and 5 display the change in the proportion of babies with birthweight <3^rd^ and <10^th^ centile respectively at preterm, early term and late term gestations over time and by birthweight centile chart. The decrease in the proportion of babies with birthweight <3^rd^ centile and <10^th^ was greatest at term gestations.

**Table 4:** Proportion (%) of babies born with birthweight <3^rd^ centile at preterm, early term and late term gestations in each two-year epoch by birthweight centile chart.

		1983–84	1993–94	2003–04	2013–14	2016–17
**Number of babies**	**34–36 weeks’**	4310	4486	5218	7154	7567
	**37–39 weeks’**	40069	51765	58365	84097	93857
	**40–41 weeks’**	67173	61502	54974	56612	50597
		**Proportion with birthweight <3**^**rd**^ **centile (%)**				
**Roberts et al. (1991–1994)**	**34–36 weeks’**	4.2	3.1	3.8	3.3	2.8^a^
	**37–39 weeks’**	3.5	3.1	2.8	2.2	1.8^a^
	**40–41 weeks’**	3.5	3.1	2.4	1.8	1.5^a^
**Dobbins et al. (1998–2007)**	**34–36 weeks’**	3.9	3.1	3.7	3.2	2.7^a^
	**37–39 weeks’**	3.8	3.4	3.0	2.4	2.0^a^
	**40–41 weeks’**	3.9	3.4	2.7	2.1	1.7^a^
**AIHW (2004–2013)**	**34–36 weeks’**	4.0	3.2	3.9	3.4	2.9^a^
	**37–39 weeks’**	4.3	3.9	3.4	2.7	2.3^a^
	**40–41 weeks’**	4.3	3.9	3.1	2.5	2.0^a^
**INTERGROWTH-21**^**st**^	**34–36 weeks’**	4.5	3.6	4.3	3.8	3.5^b^
	**37–39 weeks’**	2.1	1.9	1.7	1.2	0.9^a^
	**40–41 weeks’**	1.7	1.4	1.1	0.8	0.5^a^

^a^ P-values < 0.001 (1983–84 proportion vs. 2016–17 proportion)

^b^ P-values < 0.05 (1983–84 proportion vs. 2016–17 proportion)

**Table 5:** Proportion (%) of babies born with birthweight <10^th^ centile at preterm, early term and late term gestations in each two-year epoch by birthweight centile chart.

		1983–84	1993–94	2003–04	2013–14	2016–17
**Number of babies**	**34–36 weeks’**	4310	4486	5218	7154	7567
	**37–39 weeks’**	40069	51765	58365	84097	93857
	**40–41 weeks’**	67173	61502	54974	56612	50597
		**Proportion with birthweight <10**^**th**^ **centile (%)**				
**Roberts et al. (1991–1994)**	**34–36 weeks’**	12.3	11.0	11.8	11.7	12.3
	**37–39 weeks’**	11.3	10.1	9.3	8.5	8.2^a^
	**40–41 weeks’**	11.2	9.8	8.3	7.3	6.6^a^
**Dobbins et al. (1998–2007)**	**34–36 weeks’**	11.5	9.8	10.9	10.8	11.2
	**37–39 weeks’**	12.0	10.7	9.9	9.1	8.7^a^
	**40–41 weeks’**	12.6	11.2	9.5	8.4	7.5^a^
**AIHW (2004–2013)**	**34–36 weeks’**	11.8	10.2	11.2	11.2	11.4
	**37–39 weeks’**	12.8	11.4	10.5	9.8	9.4^a^
	**40–41 weeks’**	13.3	11.8	10.1	9.0	8.0^a^
**INTERGROWTH-21**^**st**^	**34–36 weeks’**	11.5	10.1	11.0	10.9	11.5
	**37–39 weeks’**	6.9	6.2	5.6	5.0	4.6^a^
	**40–41 weeks’**	6.2	5.5	4.5	3.8	3.1^a^

^a^ P-values < 0.001 (1983–84 proportion vs. 2016–17 proportion)

## Discussion

Here we confirm significant differences between each updated population-based Australian birthweight centile chart when applied to the same population. The proportion of babies with birthweight <3^rd^ and <10^th^ centile was recalibrated in each updated version, progressively changing the definition of small over time. In comparison, the external prescriptive INTERGROWTH-21^st^ birthweight standard yielded a substantially lower proportion of babies with birthweight <3^rd^ and <10^th^ centile than the population birthweight charts. For the Australian population, these observations suggest that population-based descriptive birthweight centiles are superior to externally-derived prescriptive birthweight standards, but should not be updated over time.

The intended purpose of updating population birthweight charts is to identify trends in population characteristics over time. The literature is rich in its explanation of birthweight trends [16], mostly attributing them to increasing rates of maternal obesity and falling rates of smoking [17–20]. The Australian birthweight centile charts have been updated twice over the last 20 years in recognition of these changes. Recalculating the birthweight centiles each decade to account for changes in population characteristics ensures that the charts remain an accurate descriptive representation of the population. However, it was recently reported that the effect of increasing intervention preferentially removing smaller babies from later gestations is likely to be the primary driver for shifting birthweight centiles [3]. Our observations in this study confirm this, illustrating that the proportion of births <3^rd^ and <10^th^ centile have decreased over time in each birthweight chart. That the effect was primarily at term gestations and not preterm gestations suggests that the shifting of centiles is an artefactual effect of increasing intervention, as previously suggested [3], rather than true biological increases in fetal size as a result of changing maternal characteristics.

Though updating birthweight centiles over time is useful in identifying these trends, it causes a problem where updated charts are used to audit care. Progressively increasing the birthweight cutoffs that define the lower centiles changes the definition of small and progressively includes more normally grown babies in the <3^rd^ and <10^th^ centile groups. When audit of care switches from using an old birthweight centile chart to an updated chart, clinicians will find that their “performance” in the detection of small babies deteriorates. This will drive them to intervene more at a higher birthweight in an effort to improve detection and hospital performance. This will increase the birthweight cutoffs further and give rise to a self-fulfilling prophecy of ever-increasing intervention. The inherent problem with updating population-based centiles and using them to audit care is that there will always be a smallest 3% or 10%. This flaw compromises the application of these charts when used to audit care and hospital performance.

A possible solution is to use a prescriptive birthweight standard, such as those produced by INTERGROWTH-21^st^, to audit care. However our findings, and those of others [21–25], give reason to be cautious of this approach too. We found that the normative INTERGROWTH-21^st^ birthweight distribution is left-shifted compared to Australian population centiles, yielding a much lower proportion of babies with birthweight <3^rd^ and <10^th^ centile. While this will identify the most severely growth restricted infants, thereby minimising the number of constitutionally small babies within the lower centiles, it will come at the expense of missing many growth restricted babies at high risk of adverse outcome [26, 27]. We also showed that, when using a prescriptive birthweight standard, the effect of targeted earlier delivery of FGR led to almost total elimination of babies with birthweight <3^rd^ centile over time at late term gestations. For these reasons, we believe that INTERGROWTH-21^st^ birthweight centiles should not be used to audit care in an Australian population. While it may be useful for international comparisons, as a standard for audit of care it is inferior to population-derived charts. Other nations, such as New Zealand [4], who do use INTERGROWTH-21^st^ centiles assess their appropriateness for the population, if they have not already done so.

Some have suggested that customizing birthweight centiles for maternal characteristics, such as maternal height, weight and ethnicity, might better identify growth restricted babies that are at high risk of adverse outcome–the principal aim of these charts [23, 25, 28]. However, the key assumption underlying customized charts–that the maternal factors are physiological and not pathological–is fundamentally flawed [29–31]. Studies supporting the use of customized centiles also include preterm gestations, where maternal characteristics contribute little information [29]. The widespread uptake in the United Kingdom of customized growth charts and associated education does not plausibly explain recent reductions in stillbirth [32].

For the purposes of audit of care, our findings recommend that jurisdictions simply not change charts over time. The advantage of this is that changes in the population can be followed. A falling proportion of babies with birthweight <3^rd^ or <10^th^ centile over time is representative of improved care, not bigger babies. It is a feature, not a bug, of birthweight centile charts, the result of better detection and timely delivery of FGR before the fetus crosses critical centiles. This needs to be recognised in jurisdictions that drive improved detection of FGR and judge performance based on a birthweight centile outcome, such as that which is done in Australia, New Zealand, and the UK [4–7]. Here the proportion of babies <3^rd^ or <10^th^ centile is likely to fall over time due to better detection–the principle aim of hospital performance reporting. If the charts are updated, causing the birthweight cutoff defining the 3^rd^ and 10^th^ centile to artificially increase, it will always be unclear how much the lower centiles have been diluted with normally grown babies. To that end, we propose that in Australia, the Dobbins et al. chart [13], which is already in widespread use, should continue to be the standard used for the audit of care.

Of course, part of the problem is that we are using a postnatal birthweight measure to judge antenatal decisions that use ultrasound-derived estimated fetal weight centiles. Updating population-based birthweight centiles will lead to increasing drift between the birthweight centiles used to audit care and ultrasound-derived centiles used to make clinical decisions [3]. With a centile distribution alone, there will always be a tradeoff between sensitivity and specificity. The broader goal, then, is to audit care not by size alone [33]. Clinicians need a standard, or markers of actual pathology [34–36], that identify those babies who will benefit from earlier delivery. Audit of care can then use those markers to measure clinical performance.

There were some limitations in this analysis. We could not examine the impact of changes in maternal characteristics, such as increasing body mass index and falling rates of smoking, on birthweight centiles because we did not have these data for all included epochs. Dating of gestational age has also changed in the VPDC. In the early epochs last normal menstrual period was principally used to determine gestation, but this has increasingly been replaced by early pregnancy ultrasound dating over time. The average effect of this in changing estimated gestation is less than a week [37] and so would not be expected to significantly alter our findings.

## Conclusion

Both population-based and prescriptive birthweight centile charts have significant flaws when used to audit care. In an Australian population, population-based charts still appear superior. If they continue to be used for the purposes of clinical audit, they should not be changed over time.
